# Defect Detection in Arc-Welding Processes by Means of the Line-to-Continuum Method and Feature Selection

**DOI:** 10.3390/s91007753

**Published:** 2009-09-29

**Authors:** P. Beatriz Garcia-Allende, Jesus Mirapeix, Olga M. Conde, Adolfo Cobo, Jose M. Lopez-Higuera

**Affiliations:** Photonics Engineering Group, University of Cantabria, Avda. de los Castros S/N, 39005 Santander, Spain; E-Mails: jesus.mirapeix@unican.es (J.M.); olga.conde@unican.es (O.M.C.); adolfo.cobo@unican.es (A.C.); miguel.lopezhiguera@unican.es (J.M.L.-H.)

**Keywords:** arc-welding, plasma spectroscopy, feature selection, on-line monitoring

## Abstract

Plasma optical spectroscopy is widely employed in on-line welding diagnostics. The determination of the plasma electron temperature, which is typically selected as the output monitoring parameter, implies the identification of the atomic emission lines. As a consequence, additional processing stages are required with a direct impact on the real time performance of the technique. The line-to-continuum method is a feasible alternative spectroscopic approach and it is particularly interesting in terms of its computational efficiency. However, the monitoring signal highly depends on the chosen emission line. In this paper, a feature selection methodology is proposed to solve the uncertainty regarding the selection of the optimum spectral band, which allows the employment of the line-to-continuum method for on-line welding diagnostics. Field test results have been conducted to demonstrate the feasibility of the solution.

## 1. Introduction

The fields where welding processes are key factors in the production stages cover a wide range of different applications: manufacturing of pipes for various sectors, engines for aeronautics, automobiles or heavy components for nuclear power stations are some relevant examples in this regard. The lack of a complete comprehension of the physical phenomena occurring during the welding process, and the demanding quality standards to be found in this framework, have forced scientists to carry out an intense research effort in both welding physics and procedures devoted to cope with quality issues. Some of these studies have been focused on the development of theoretical models for both arc and laser welding [1-3], including numerical analysis approaches [4]. These efforts help to understand the process and, therefore, to determine the precise input parameter ranges that will provide seams free of flaws.

However, in practice welding coupons employed for parameter adjustment, and both destructive and non-destructive trials [5] have to be used to ensure that the performed seams satisfy the established quality standards. This obviously implies a significant cost in terms of productivity, as a lot of time is spent before and after the welding process itself, and, therefore, some of the seams have to be reworked and evaluated again.

This scenario has led to an intense research effort aimed at developing efficient and reliable on-line welding quality monitoring systems. They should be able to detect in real-time the occurrence of possible defects and, as an added value, to control the welding setup to try to avoid these defects or drifts from the standard operation conditions. Several techniques have been proposed, from electrical and capacitive sensors [6,7], to monitoring based on the analysis of the acoustic signal generated during the process [8,9] or solutions based on machine vision [10,11]. Among these alternatives, the optical analysis of the welding plasma radiation has proved to be a feasible and promising option. Initial proposals were based on the use of photodiodes and the analysis of emissions in the ultraviolet, visible and infrared regions [12], determining for example the full-penetration condition in laser welding [13].

A more sophisticated approach has been proposed by considering plasma optical spectroscopy, where emission lines appearing in the plasma spectra are analyzed to provide a plasma electron temperature *T_e_* profile that shows a direct correlation to weld defects [14,15]. In the last years, several publications have dealt with refinements of this technique, allowing automatic defect detection [16] and reducing the overall computational cost of the system [17]. More recently, new strategies have been proposed to extract more information from the plasma spectra, like the correlation analysis proposed by Sibillano *et al.* [18], or proposals based on the use of optimization algorithms to generate synthetic spectra [19]. Within the same framework, new spectroscopic parameters are also being studied in an attempt to improve the monitoring system efficiency [20].

One of the key issues when using plasma spectroscopy lies in the correct selection of the emission lines chosen to calculate the output monitoring parameter. On the one hand, and depending on the selected instrumentation, there can be ambiguities on the emission line identification, what can end in unexpected results. On the other hand, and especially when defect classification is required, i.e., to be able to distinguish among different types of defects, it would be highly interesting to know which emission lines allow a better discrimination for classification purposes.

We have conducted some previous studies by using PCA (Principal Component Analysis) and SFFS (Sequential Forward Floating Selection) to feed an Artificial Neural Network [21,22]. The use of SFFS allows to gain knowledge about the best spectral bands selected. This will be used in this paper to propose a scheme based on both the SFFS algorithm and the line-to-continuum method [23] to generate the required output monitoring profiles. The line-to-continuum method implies the use of only a single emission line that, in addition, does not need to be identified, i.e., associated with its chemical species.

## 2. Plasma Optical Spectroscopy for Welding Diagnostics

The plasma electron temperature has been widely used as the output monitoring parameter for welding diagnostics, given the known correlation between its profiles and the appearance of defects in the seams. There are basically two approaches that are employed in the literature: a precise estimation of *T_e_* can be obtained with the Boltzmann-plot method [23]:

(1) $$\ln\left(\frac{I_{\text{mn}}\lambda_{\text{mn}}}{A_{\text{mn}}g_{m}}\right)=\ln\left(\frac{hcN}{Z}\right)-\frac{E_{m}}{kT_{e}}$$

where several emission lines from the same species are involved in the calculations. In the previous equation *I_mn_* is the relative intensity of the chosen emission line, *m* and *n* the upper and lower states, respectively, *λ_mn_* the central wavelength associated with the line, *A_mn_* the transition probability, *g_m_* the statistical weight, *h* the Planck's constant, *c* the light velocity, *N* the population density of the state *m, Z* the partition function, *E_m_* the upper level energy and *k* the Boltzmann constant. *T_e_* can be obtained if the left-hand side of Equation (1) is represented versus *E_m_*, given that the slope of the resulting line is inversely proportional to the temperature.

On the other hand, and due to considerations regarding the computational performance of the monitoring system, which determines its spatial resolution, a simplification of the Boltzmann-plot method, where only two emission lines are involved, is typically used:

(2) $$T_{e}=\frac{E_{m}(2)-E_{m}(1)}{k\ln\left[\frac{E_{m}(1)I(1)A(2)g_{m}(2)\lambda (1)}{E_{m}(2)I(2)A(1)g_{m}(1)\lambda (2)}\right]}$$

This equation was proposed by Marotta [24] for arc-welding processes. The commented techniques can be equally applied to both arc and laser processes, although for the latter the energies of the upper level disappear from the logarithm in the denominator.

**Figure 1.**
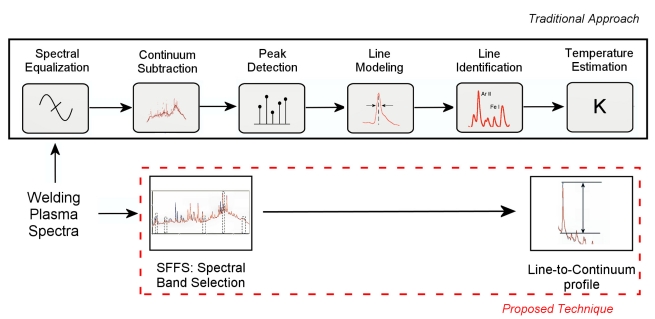
Comparison of the processing schemes associated with the traditional approach and the proposed solution based on SFFS and the line-to-continuum method.

Although different approaches can be taken into account, a possible processing scheme designed to provide the required *T_e_* estimation from the acquired welding plasma spectra is presented in Figure 1.

The identification of the emission lines is compulsory to obtain *T_e_*. As shown in Figure 1, this requires three additional processing stages (peak detection and line modeling and identification) and it has, as a consequence, a direct implication in the real-time performance of the overall approach. An alternative solution is to perform a previous spectral band selection stage with a data set consisting of spectra from the same welding process under different conditions. Afterwards those lines can be used without involving the identification in the processing scheme.

This could be applied for scenarios where the same materials and welding conditions are used, but it limits the flexibility of the analysis strategy. On the other hand, as previously commented, there is a lack of knowledge on the selection of the optimal emission lines for welding diagnostics. Some studies have been carried out comparing the response of different elements and species, but we believe that by specifically searching for the most discriminant spectral bands the overall performance of the monitoring system should be improved.

Within this framework, the use of the line-to-continuum method to generate the output monitoring profiles seems to be a good solution, given that it does not require the identification of the chosen emission line. However, it could be performed to avoid problems related to the effect of unresolved lines. This method was originally intended to estimate *T_e_* by means of the following expression [25]:

(3) $$\frac{{ɛ}_{l}}{I_{c}}(\lambda )=2.0052\times 10^{-5}\frac{A_{\text{mn}}g_{m}}{Z_{i}}\frac{1}{T_{e}\xi }\exp\left(\frac{E_{i}-E_{m}}{kT_{e}}\right)\frac{\lambda }{\Delta \lambda }$$

where *ε_l_* is the line intensity integrated over the line profile, *I_c_* the intensity of the adjacent background radiation (non-integrated), *Z_i_* is the ion partition function, *ζ* the free-bound continuum correction, *E_i_* the ionization potential and *Δλ* the wavelength bandwidth. It is worth mentioning that an iterative method has to be employed to determine *T_e_* via Equation (3). However, in the proposed method we only use the *ε_l_/ I_c_* ratio as the monitoring parameter. In a previous paper, this approach was initially explored in comparison to an alternative method based on the estimation of the wavelength associated with the maximum intensity of the continuum radiation [20]. In this case we concluded that the line-to-continuum method could not be reliably used given the uncertainty regarding the selection of the spectral band for the subsequent analysis. In this paper the use of the SFFS algorithm will help to deal with this issue.

## 3. Sequential Floating forward Selection of Spectral Bands

The Sequential Floating Forward Selection (SFFS) algorithm [26] is widely applied to reduce the dimensionality, i.e., the number of features or wavelengths, of spectral data prior to their interpretation [27,28]. The spectral band selection criterion is based on the capability of the distinct features to separate the different classes to be discriminated afterwards. The greater the separability between the classes that a wavelength provides, the better the wavelength is for classification purposes. Therefore, a similar approach could be followed to solve the uncertainty encountered in the selection of the optimum band within the plasma spectra for on-line welding quality monitoring by means of the line-to-continuum method described above [20]. The aim of SFFS is to select *M* spectral bands that best discriminate among correct welds and flaws, out of the total number of initial bands *N*, so *M* ≪ *N*. In this way the line-to-continuum profiles obtained later on for these *M* bands will be optimized for defect detection. It is worth highlighting here that SFFS has already been used in some previous works by our group [22]. However, the focus was, on the contrary, to provide a set of spectral bands to feed an Artificial Neural Network (ANN) for flaw discrimination as an alternative to the widely known feature extraction techniques [21].

As mentioned before, the selection criterion for band selection is an objective function based on a measurement of the separability of the classes. Specifically, it is estimated in terms of the Bhattacharyya distance as in [22]:

(4) $$J_{B}=\frac{1}{4}{\left(\mu_{2}-\mu_{1}\right)}^{T}\left[\sum_{1}+\sum_{2}\right](\mu_{2}-\mu_{1})+\frac{1}{2}\log\left(\frac{\left|\sum_{1}+\sum_{2}\right|}{2{\left(\left|\sum_{1}\right|\cdot \left|\sum_{2}\right|\right)}^{\frac{1}{2}}}\right)$$

where μ_i_ is the mean of the i class; Σ_i_ its covariance matrix and |Σ_i_| stands for the determinant of matrix Σ_i_ There are only two classes to be distinguished here: correct seams and defects. Therefore, the overall separability among the classes to be discriminated is straightly given by (4).

**Figure 2.**
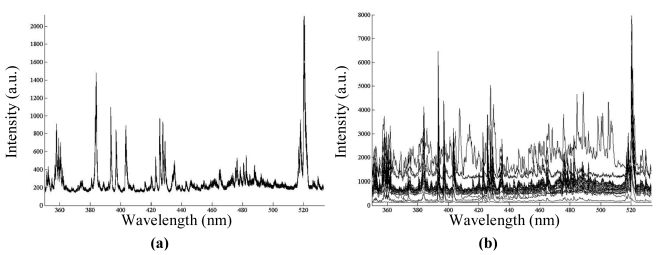
Spectral curves of: **(a)** several correct seams. **(b)** distinct flaws.

In Figure 2 several spectral curves of correct welds (a) and defects (b) are presented. As shown, some ranges of the spectra exhibit similar characteristics and, as a consequence, they are useless for defect detection purposes. The elimination of these ranges is compulsory prior to the application of SFFS. This procedure is named data decorrelation since only one spectral band, the one that maximizes the Bhatacharya distance, is selected within each redundant block. Each redundant block is obtained as a wavelength range where spectral bands have a high correlation coefficient, nearly 1.

**Figure 3.**
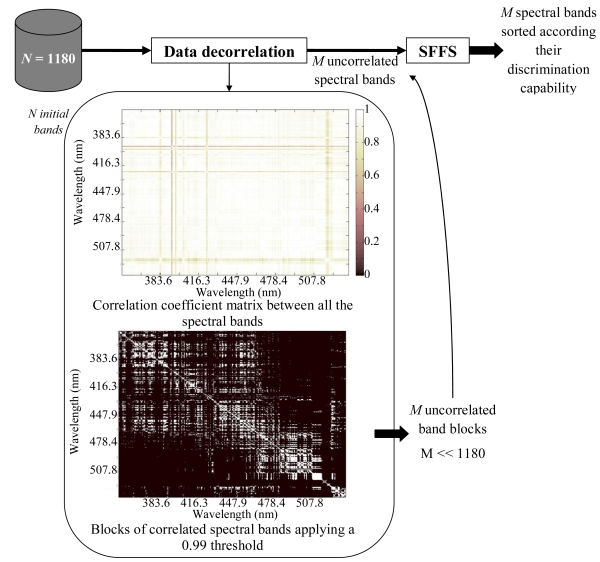
Block diagram of the spectral band selection and sorting procedure.

SFFS is named “*forward*” because it begins with an empty selected feature subset. It initially selects from the uncorrelated subset of spectral bands the most discriminant one according to the Bhattacharyya distance. Then it carries on sequentially adding the second most discriminant wavelength and so on. Therefore, in each iteration a new spectral band is added. However, if the iteration number is greater than 1, some of the previously added features can be removed if the separability of the classes enhances. Then, after every forward step (addition of a new spectral band) a number of backward steps (removal of several spectral bands) are applied as long as the resulting subsets are better than the previously evaluated ones according to the Bhatacharrya distance, and for that reason the selection procedure is also named “*floating*”. This procedure continues until some termination criterion (for example the number of maximum features to be used or admissible maximum classification error) is met. In this case, the goodness of almost all uncorrelated bands, but sorted as given by the SFFS algorithm, i.e., in terms of their flaw detection capability, will be evaluated for defect discrimination purposes. A schematic diagram of the overall band selection procedure is depicted in Figure 3.

## 4. Evaluation and Discussion of Experimental and Field Tests

Analyses of different types of weld processes have been performed to study the influence of the spectral band selection on the resulting output line-to-continuum profiles. This issue was initially explored in a previous publication by our group [20], where an alternative technique was proposed, but line-to-continuum profiles were generated to allow comparison between both approaches. It seemed clear that the obtained results exhibited strong dependence on the chosen spectral band, what gave rise to the suitability of including a selection method in this regard.

**Figure 4.**
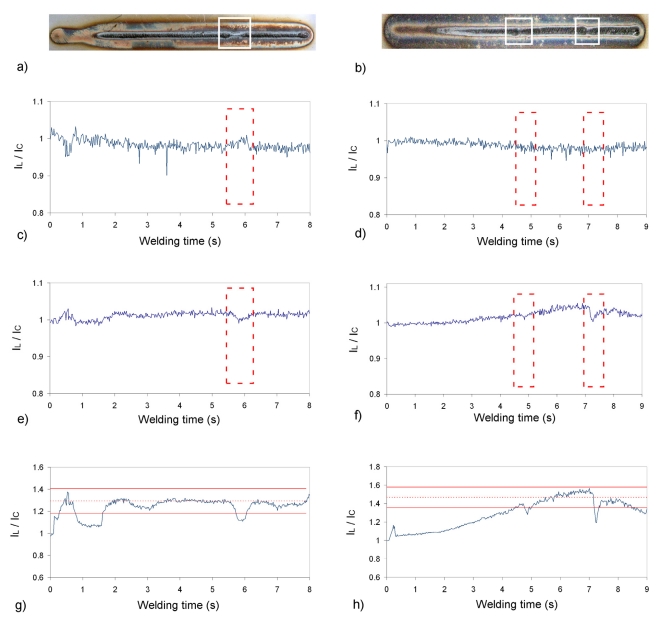
Detection of weld flaws by means of line-to-continuum method: a) and b) bead-on-plate seams with defects; c) and d) output profiles with spectral band 518.41 nm; e) and f) output profiles with spectral band 375.03 nm; g) and h) output profiles with spectral band 481.08 nm.

Initial analyses were conducted on AISI-304 stainless steel plates, performing bead-on-plate seams with a TIG (tungsten inert gas) setup. The plasma optical radiation was collected by means of a 2 m optical fiber (50 μm core diameter) attached to an Ocean Optics USB2000 CCD-spectrometer with a spectral range from 195 to 535 nm. Argon was used as shielding gas, with a flow rate in normal operating conditions of 12 L/min. Three defects were provoked on two seams by simulating a perturbation on the gas flow, manually performing a shortening to 2 L/min during approximately 0.5 s. These seams are presented in Figure 4a,b, where the defects under analysis have been highlighted. The use of the SFFS algorithm for these two experimental tests provided the set of spectral bands listed in Table 1.

It can be observed that the selected emission lines have been also identified, and precisely the first one is not associated with an emission line but to continuum. * denotes that the spectral band is not centered on a particular emission line, but it is affected by its changes, i.e., it lies in the vicinity of that line. Figure 4 shows the discrepancies to be found in the line-to-continuum profiles depending on the spectral band selected. In these profiles *I_L_* stands for the relative intensity of the chosen spectral band and *I_C_* for the corresponding background radiation.

Figure 4c,d presents the results obtained with spectral band 518.41 nm, Figure 4e,f with 375.03 nm and Figure 4g,h with 481.08 nm. The latter offers the best results in terms of defect discrimination, given that the three flaws located at x ≈ 6 cm for seam n° 1 [Figure 4a], and at x ≈ 5 and 7 cm for seam n° 2 (Figure 4b) are associated with rapid perturbations on the profiles. On the contrary, Figure 4c,d does not show any correlation with these defects, indicating that the selected spectral band is not suitable in this case. By using the spectral band located at 375.03 nm the identification of the defects is again feasible, but the signal-to-noise ratio is clearly poorer in this case. It is worth mentioning that the dip at the beginning of Figure 4g is due to the use of a lower welding current at the beginning of the process. As expected, a lower plasma electron temperature is associated with a lower welding current, given that the line-to-continuum profile is directly related to this parameter as suggested by Equation (3). The slope of Figure 4h was caused by a non-constant distance between the electrode tip and the plate provoked by the deformation of the plate by heat during previous seams. Again, the slope is associated with *T_e_*, being the temperature higher as the distance between the electrode tip and the plate becomes smaller. These two situations can be also considered as defective, although the analysis via SFFS has been performed considering only the sections highlighted in Figure 4a,b as weld defects.

An example of automatic weld defect detection has been included in Figure 4g, using the approach proposed by Ancona *et al.* in [16]. In this case the reference signal has been generated considering the seam section between x ≈ 3.5 and x ≈ 5.5 cm as correct. The corresponding thresholds have been plotted by determining both the *I_L_/I_C_* mean and standard deviation values, and chosing *α* = 10 [16]. It can be observed that not only the defect at x ≈ 6 cm, but also the lack of penetration at the beginning of the seam would be identified as defective, as the monitoring signal exceeds the calculated thresholds. The same analysis has been conducted in Figure 4h, where again the signal associated with the defects under analysis and the initial section of the seam exceeds the thresholds.

The results related to spectral bands 375.03 and 518.41 nm are worse than expected taking into account that their selection by the SFFS algorithm is performed earlier than the selection of the 481.08 nm spectral band. An initial analysis indicates that a possible explanation lies in the suppression of bands correlated to these ones that would offer better correlation to the quality of the seams. This occurs for example between spectral bands located at 375.03 and 446.07 nm. The latter gives rise to profiles similar to the ones depicted in Figure 4g,h, but this band is correlated to the former and, consequently, suppressed by the SFFS algorithm.

**Figure 5.**
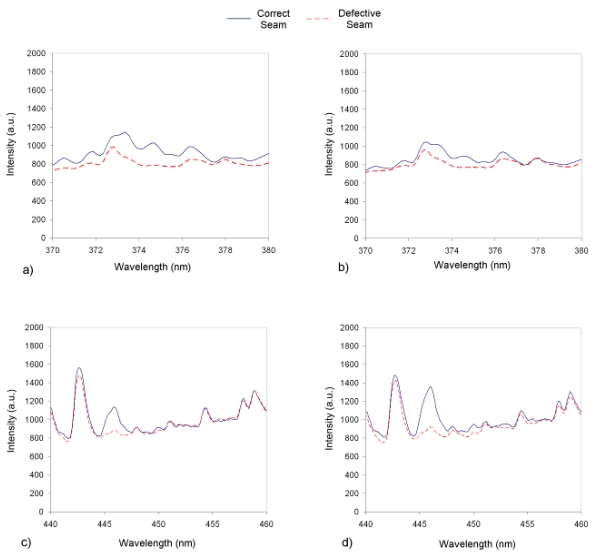
Comparison of spectra associated with correct and defective seams: a) and b) spectral band 375.03 for seams n° 1 and 2; c) and d) spectral band 446.07 for seams n° 1 and 2.

The plasma spectra for both experimental tests and spectral bands have been represented in Figure 5. It can be appreciated how the band chosen by SFFS does not belong to an emission line, while in 446.07 nm a significant peak is to be found.

A feasible explanation for the uncertainty in the SFFS sorting criterion of the spectral bands is based on its assumptions about the probability distribution functions of the data. The employment of the SFFS algorithm implies a Gaussian or normal distribution of the classes. Correct welding could fit with this assumption but this is not necessarily the case of the defect class taking into the account the complexity of the physical phenomena occurring during the processes. An easy way to estimate whether the class distributions are Gaussian or not is by evaluating its third and fourth order statistics moments, namely skewness and kurtosis [29].

**Figure 6.**
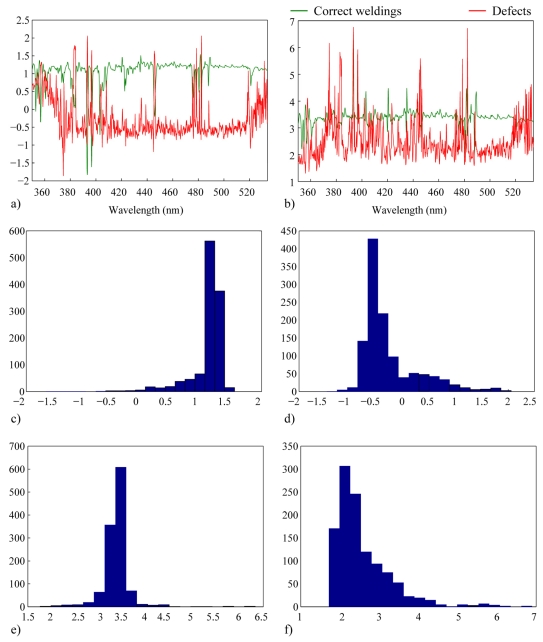
Modelling of the data distribution function of the classes: a) and b) skewness and kurtosis values per class and wavelength; c) histogram of skewness values of correct weldings d) histogram of skewness values of defects; e) and f) idem for kurtosis values.

Skewness is a measure of the asymmetry of the data around the class mean. Therefore the skewness of the normal distribution (as in any other perfectly symmetric distribution) is zero. Kurtosis is a measure of how outlier-prone a class distribution is and the kurtosis of the normal distribution is 3. Skewness and kurtosis values per class and wavelength are displayed in Figure 6a,b, respectively, while Figure 6c-f exhibits their corresponding histograms. Neither the skewness histogram pertaining to the correct welding class nor the one pertaining to defects are centered around zero, which allows us to conclude that none of the classes have a normal distribution. In spite of this fact, SFFS provides the selection of some spectral bands that allow defect detection such as the one in 481.08 nm and, as a consequence, a two step methodology is proposed. First the SFFS algorithm is applied and the line-to-continuum profiles are obtained for the selected wavelengths. Then the signal-to-noise ratio of these profiles is evaluated and the one with the highest value is selected as the monitoring signal.

The reordering of the spectral bands in terms of the S/N* (Defect Sensitivity) parameter is presented in Table 2. A detailed study of the resulting line-to-continuum profiles was conducted to elucidate a suitable method to quantify their sensitivity to detect the weld flaws. This S/N* is calculated as follows: first, the signal-to-noise ratio over a section free of defects is estimated (*S/N* in Table 2), being in this case the seam segment between welding times 3 and 5 s for seam n° 1. Afterwards, the defect detection sensitivity is considered as the difference between the *I_L_/I_C_* value at the defect (x ≈ 6 s) and x = 5.5, where the signal indicates a correct seam. Finally, this two values are multiplied to generate the S/N* parameter. This new ordering of the spectral bands is in good agreement with the results shown in Figure 4, as the spectral bands corresponding to Figure 4c,e,g is classified with this new method in 19^th^, 16^th^ and 4^th^ positions.

The proposed technique has been also checked with data obtained during field tests of a tube-to-tubesheet orbital TIG welding process. The material to be welded was INCONEL 690 tubes, and the tests were developed on a welding coupon simulating the conditions of a steam generator for a nuclear power station. The process is described in detail in [30].

**Figure 7.**
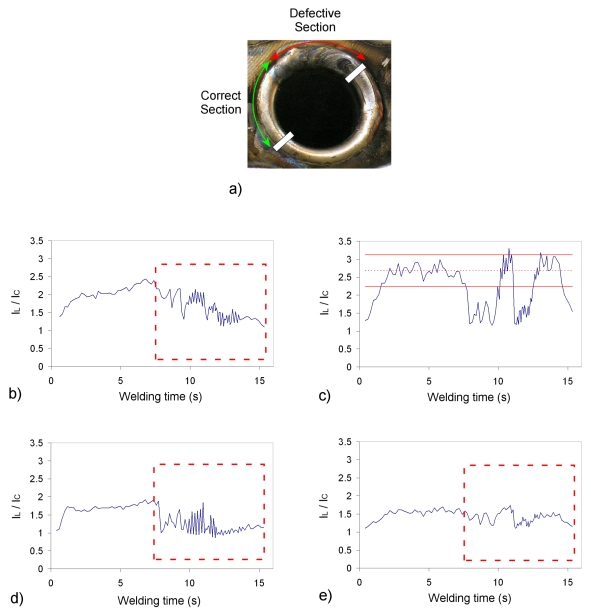
Field test tube with defective section: a) welded tube; b) profile for spectral band 404.14 nm; c) profile for spectral band 422.84 nm; d) profile for spectral band 480.52 nm; e) profile for spectral band 423.64 nm.

As the material and process parameters were completely different from the previously discussed experimental tests, a new set of spectral bands had to be generated with SFFS. The new bands are displayed in Table 3, and Figure 7 depicts the chosen output profiles for a tube with a defective section. The S/N* parameter has been included in the Table, being in this case estimated with the field test shown in Figure 7. The flaw was provoked by a perturbation on the gas flow rate by the operator, what gave rise to the defective section highlighted in Figure 7a. It can be appreciated that the defective section is associated with perturbations on the *I_L_/I_C_* profiles, while the seam labelled as correct is more stable. The perturbation on the protection gas flow is reflected on the welding plasma, and consequently also on the intensity of the different emission lines versus the continuum radiation. The operator catalogued the seam as defective after visual inspection, given the irregularities to be observed on the surface, as well as the associated bead color. Further analyses by means of destructive or non-destructive evaluation techniques could have helped to improve this classification, but in this case visual inspection was considered to be adequate. According to the ISO 6520-1:1998 standard this defect could be labelled as an irregular surface (excessive surface roughness). Four different spectral bands have been considered in this case: 404.14, 422.84, 480.52 and 423.64 nm. It is precisely the latter, associated with Fe I, the one which exhibits worst results in terms of sensitivity to defects, as all the profiles have been represented with the same vertical scale. It is important highlighting that the defective section could be identified, at least partially, in the four cases, but the profile in Figure 7c offers the best sensitivity: in terms of S/N* the spectral bands are classified as 7^th^ (404.14 nm), 2^nd^ (422.84 nm), 8^th^ (480.52 nm) and 10^th^ (423.64 nm). The *I_L_/I_C_* thresholds have been calculated again for Figure 7c, using in this case *α* = 3.

**Figure 8.**
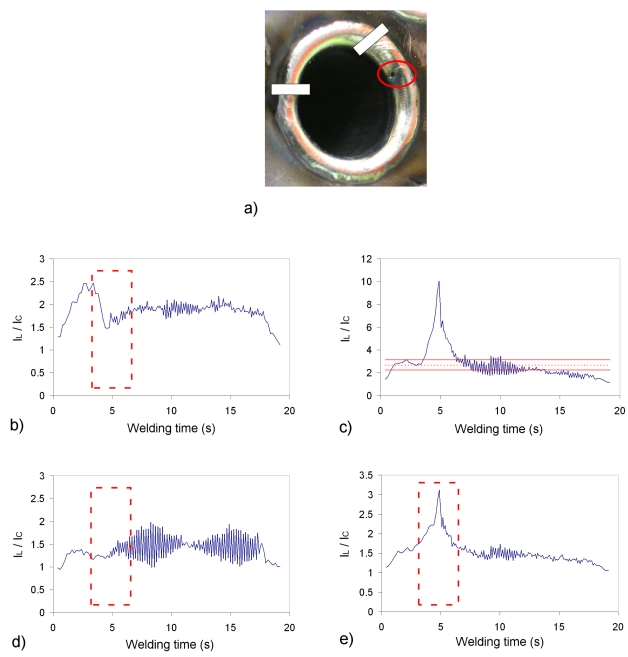
Field test tube with porosity: a) welded tube; b) profile for spectral band 404.14 nm; c) profile for spectral band 422.84 nm; d) profile for spectral band 480.52 nm; e) profile for spectral band 423.64 nm.

A different defect is presented in Figure 8a, where a porosity (highlighted in red) was created due to the application of a gel to the tube-to-tubesheet interface. It is interesting to note that, although only the porosity appears as visible, the gel was applied trough the whole interface, what probably provoked deviations from the standard quality requirements. This can be the explanation to the fast perturbations that appear in the profiles of Figure 8b-e. Again, Figure 8c, where the *I_L_/I_C_* thresholds have been plotted, offers the best identification of the porosity and the higher defect sensitivity. It is interesting to note that in this case the classification provided by S/N* seems valid for the first three bands, but the fourth (Figure 8e) exhibits a better response than Figure 8b, for example. This can be explained because the S/N* analysis was performed with the field test associated with Figure 7. In this case the generalization of these classifications to both seams would not be valid, and a separate study should be conducted.

## 5. Conclusions

A spectroscopic approach based on feature selection and the line-to-continuum method for on-line detection of welding defects is proposed and experimentally validated in this paper. Compared with the determination of the plasma electron temperature, the main advantage of the line-to-continuum method is its computational efficiency, since it does not imply the identification of the atomic emission lines. However, its defect detection capability highly depends on the chosen emission lines. Therefore, an initial stage is required to determine those spectral bands from the welding plasma that best discriminate among correct welding and the appearance of defects. In a previous work [22] the SFFS algorithm was employed to reduce spectral dimensionality of data from an embedded fiber-sensor for on-line welding diagnostics. Selection criterion of the spectral bands was based on the maximization of the distance between the different flaws and correct welds. This same approach has been applied here to get rid of the uncertainty regarding the selection of the optimum spectral bands. SFFS is firstly utilized to identify the proper lines for defect detection. Then, the monitoring signal consists of the line-to-continuum profiles of those lines. Experimental results show a strong correlation between the appearance of flaws and abrupt changes in the monitoring signal.

The conducted studies, which include an extended set of defects with different target materials, have demonstrated the suitability of the methodology for on-line analysis. However, they have also brought up that some uncertainty still remains regarding the SFFS sorting criterion of the spectral bands. As shown, it occurs that some of the spectral bands that are selected later on by SFFS have proved more successful in defect-detection. This is due to the fact that the wavelength selection criterion, the Bhatacharyya distance, assumes a Gaussian distribution of the classes and neither correct weldings nor the defect class fit with this assumption. As a consequence, a further comparative study on the capability of the selected wavelengths for flaw detection has been performed, being the signal-to-noise ratio of the line-to-continuum profiles the comparison criterion. Regarding the use of the S/N* parameter to perform this fine classification of the spectral bands, it would be interesting to define a more general approach able to quantify the sensitivity to the appearance of different defects. Current studies are on going focused on this objective and also on the classification of the defects in order to actuate on the precise welding parameter to try to prevent each defect from happening. It should be also investigated if a set of different spectral bands is needed to accomplish discrimination between different defects, or an approach based in a single band is feasible, even to be used for different materials and processes.

## Figures and Tables

**Table 1.** Spectral bands selected and listed by SFFS and related to species participating in the plasma: experimental tests.

**Spectral band (nm): order**	**Species**	**Spectral band (nm): order**	**Species**	**Spectral band (nm): order**	**Species**

375.03: (1)	Cont.	357.93: (9)	Cr I	393.04: (17)	Cr I *
518.41: (2)	Cr I	396.48: (10)	Cr I	488.14: (18)	Ar II *
392.87: (3)	Fe I	403.33: (11)	Mn I	382.00: (19)	Fe I
481.08: (4)	Mn I *	396.64: (12)	Cr I	355.74: (20)	Ni I
434.75: (5)	Cr I	435.70: (13)	Cr I *	357.09: (21)	NI I *
480.62: (6)	Ar II	439.95: (14)	Fe I	423.00: (22)	Ar II *
487.84: (7)	Ar II	397.13: (15)	Cr I *	356.75: (23)	Ni I
360.63: (8)	Cr I	357.26: (16)	Cr I *		

**Table 2.** Spectral bands selected and listed by SFFS and reordered by S/N Defect Sensitivity.

**Spectral band (nm): SFFS-order**	**Species**	**S/N**	S/N * Defect Sensitivity (order)

403.33: (11)	Mn I	55.69	18.93 (1)
357.09: (21)	NI I *	66.91	18.73 (2)
357.26: (16)	Cr I *	74.67	16.43 (3)
481.08: (4)	Mn I *	80.5	15.22 (4)
446 (**)	Mn I	54.69	12.03 (5)
382.00: (19)	Fe I	101.71	10.17 (6)
393.04: (17)	Cr I *	19.38	9.69 (7)
356.75: (23)	Ni I	30.89	9.27 (8)
357.93: (9)	Cr I	45.94	9.19 (9)
396.64: (12)	Cr I	22.19	8.87 (10)
360.63: (8)	Cr I	58.07	8.71 (11)
392.87: (3)	Fe I	24.23	7.95 (12)
396.48: (10)	Cr I	25.16	7.55 (13)
397.13: (15)	Cr I *	37.41	7.48 (14)
423.00: (22)	Ar II *	71.84	7.18 (15)
375.03: (1)	Cont.	197.81	5.14 (16)
355.74: (20)	Ni I	36.6	4.39 (17)
487.84: (7)	Ar II	33.51	2.01 (18)
518.41: (2)	Cr I	85.16	1.79 (19)
435.70: (13)	Cr I *	89.29	1.79 (20)
480.62: (6)	Ar II	26.07	1.56 (21)
488.14: (18)	Ar II *	37.68	1.13 (22)
434.75: (5)	Cr I	27.55	0 (23)
439.95: (14)	Fe I	41.51	0 (24)

**Table 3.** Spectral bands selected and listed by SFFS and related to species participating in the plasma: field tests.

**Spectral band (nm): (SFFS-order)**	**Species**	**S/N**	S/N * (order)

516.79 (12)	Fe I	29.1	41.03 (1)
422.84 (3)	Ar II	16.1	24.30 (2)
415.95 (2)	Ar I	20.96	20.54 (3)
402.83 (6)	Mn I *	30.68	19.68 (4)
426.35 (5)	Cr I *	28.42	15.92 (5)
407.39 (9)	Cont.	42.69	14.94 (6)
404.14 (1)	Mn I *	13.91	14.47 (7)
480.52 (7)	Ar II	20.24	14.37 (8)
443.54 (11)	Fe I	28.57	14.00 (9)
423.64 (4)	Fe I	26.1	11.48 (10)
478.28 (8)	Mn I	26.28	11.30 (11)
432.28 (14)	Cont.	36.9	7.38 (12)
427.30 (10)	Cr I	52.77	3.69 (13)
520.05 (13)	Cr I	19.1	1.53 (14)
